# The Public Health Service as a Career for Medical Men

**Published:** 1908-09-05

**Authors:** Alfred Greenwood

**Affiliations:** Medical Officer of Health for Blackburn.


### THE PUBLIC HEALTH SERVICE AS A CAREER FOR MEDICAL MEN.

By ALFRED GREENWOOD, M.D., B.Sc., D.P.H., Medical Officer of Health for Blackburn.

The rapid progress and developments which have been made during recent years in preventive medicine deserve very careful attention and consideration on the part of those who have completed their medical curriculum, and also on the part of those who are about to enter the various medical schools of this country.

At the present time large numbers of medical men are devoting all their time as medical officers of health to county councils, county boroughs, municipal boroughs, and combinations of rural districts. This number is steadily increasing every year, as more appointments are constantly being created. Indeed, the large number of vacancies for whole-time medical officers of health and assistant medical officers of health which have been advertised in the medical journals already this year, have established a record. Also it may be mentioned here that, in connection with the "Housing of the Working Classes" problem, very strong recommendations have been made that there shall be a medical officer of health for every county in England. It is very likely that these recommendations will be adopted at no distant date.

The amount of work and the responsibility which have been placed upon medical officers of health have increased enormously in recent times. As an illustration of this statement it is only necessary to mention the additional duties which have been placed upon these officials in connection with the Factory and Workshop Act 1901, the Midwives Act 1902, and the Medical Inspection of School Children, which was placed upon a sound basis last year.

It is now generally recognised that the public health service has many attractions. This is shown by the number of medical men engaged in general practice who are anxious to relinquish that work and devote their whole time to public health. Amongst the attractions of the public health service may be mentioned the absorbing, varied, and scientific life which it affords, the large number of interesting problems which are still far removed from solution, the more regular hours of work, the freedom from bad debts, and the smaller expenditure which is necessary as compared with the life of a general practitioner. Also the whole-time medical officer of health is able to take his annual holiday of three or four weeks without the expense of paying a *locum*. In many cases the remuneration of medical officers of health is not high, but it is regular, and is not affected injuriously by depression of trade and so forth. Again, there is always the opportunity of obtaining larger appointments from the smaller ones, especially if the work is carried out with enthusiasm and energy. Many decided and authoritative opinions have been expressed that the minimum salaries of whole-time medical officers of health should be £500 per annum, and practical effect has been given to these opinions in several instances. There are now many whole-time medical officers of health in this country who receive salaries between £500 and £1,000 per annum.

Amongst the disadvantages of the Public Health Service may be mentioned a lack of appreciation of the medical officer's work by members of the governing body. When this occurs it is very unfortunate and discouraging, but it is not common. Also, as a rule, it is not possible for an official to amass great wealth in the Service, and in a very large number of cases he has no security of tenure of office or any superannuation fund from which he may derive benefit in the future. Nevertheless, it does seem that the advantages of the Public Health Service as a career for medical men far outweigh the disadvantages.

The public health branch of medicine is concerned with groups of people rather than with individuals. One of the chief duties of a medical officer of health is to keep himself informed of all the conditions which may affect injuriously the health of the people in his district. Consequently it is necessary for him to have some knowledge of a number of subjects, such as physics, chemistry, bacteriology, engineering, architecture, meteorology, epidemiology, etc.

In former days it was thought by many people that the work of a medical officer of health was dry and uninteresting, and that his chief duty consisted in preparing reports, the main feature of which was long columns of figures. Also it was considered by many that this official rapidly lost all interest and experience in clinical work, and that he became a kind of business man, going to and from his office daily, signing letters, and superintending inspectors of nuisances. This narrow idea no longer prevails in the minds of those who have followed, even cursorily, the work carried out by various health departments in our country. At present there is ample scope for clinical work, in the general control of the spread of infectious diseases, in the supervision of a fever hospital, and in the medical inspection of school children.

It is not possible, as a rule, for a medical officer of health to show the results of his work in pounds, shillings, and pence, as is the case with certain other municipal officials. Most of his work consists in "casting bread upon the waters." At the same time many of these health officials are able to point to definite results, as in the prompt control of a small-pox epidemic or in the successful search for the cause of an outbreak of food-poisoning. More indirect beneficial results may be shown from steady improvements in general sanitation, which, when adopted continuously in any given town, have clearly resulted in a reduction in the death-rate. In fact, it is no exaggeration to state that whereas surgery and medicine have saved thousands of lives, that special branch of medicine—namely, public health—has saved tens of thousands of lives.

It is obvious, of course, that the medical officer of health cannot perform his work successfully without considerable assistance. He must have the loyal and regular help of a staff of inspectors, clerks, and disinfectors. Also no small element of success in controlling the spread of infectious diseases lies in the existence of harmonious co-operation between the medical officer of health and his brother practitioners. The former is often called into consultation by the latter, and there are numerous ways in which the latter can help the former.

It is well known that the medical members of the public health service are divided into two groups—namely, part-time medical officers of health, who are usually engaged in general medical practice at the same time, and also medical officers of health who are devoting their whole time to this special work. For a large number of appointments an additional qualification is necessary—namely, a diploma in Public Health (D.P.H.). Every medical practitioner who wishes to gain an appointment as medical officer of health in any county, district, or combination of districts with a population of 50,000 or upwards should be registered as the holder of a diploma in sanitary science, public health, or State medicine, under Section 21 of the Medical Act, 1886, or have during 1889, 1890, and 1891 been medical officer of health of a district or combination of districts with a population of 20,000 at least; or have for three years previous to August 13, 1888, been a medical officer or inspector of the Local Government Board. Great stress is laid upon the possession of such a diploma in the Memorandum of the Board of Education which was issued in December 1907, on "The Medical Inspection of Children in Public Elementary Schools."

It is probable that in the near future important additions will be made to the work and responsibilities of medical officers of health. In illustration of this point, mention may be made of a possible reorganisation of Poor-law administration, the medical inspection of children who are leaving school, in connection with the duties of certifying factory surgeons, the possible extension of the notification of infectious diseases so as to include infantile diarrhœa, cancer, pneumonia, etc. There are some who even predict the early advent of a State medical service, to include treatment of individual illness.

There is no doubt that the general public are interested more, at the present time, than ever they were in many public health questions. Every year greater willingness is shown by people in allowing sanitary authorities to disinfect rooms after deaths from phthisis. The "man in the street" now shows an intelligent interest in such questions as the sanatorium treatment of phthisis, and is thoroughly alive to the necessity for a clean milk supply, pure water, efficient drainage, thorough meat inspection, the control of infectious diseases, etc. He knows well that all these measures have a most important effect upon his every-day life.

Such conditions certainly indicate greater enlightenment in the public mind as compared with former days.

It will be clear that it is often desirable that general medical practitioners should obtain a diploma in Public Health. This can be done at the same time that the practitioner is conducting his practice, if he happens to be within easy reach of a University town. There are so many prospects, perhaps not realised at the time, that the possession of this diploma might prove most useful. Also the advantages should be placed clearly before each medical student of proceeding to obtain the diploma in Public Health immediately after he has qualified as a general medical practitioner. It is possible to obtain this diploma twelve months after qualifying, and the necessary attendances at a laboratory can often be made during the tenure of a hospital appointment. Such a course of post-graduate work is of immense value to any medical man. The bacteriological part of the course renders his general medicine, surgery, and midwifery far more interesting and useful, and his general knowledge receives most practical additions. And, as has been hinted at above, the medical man who possesses a diploma in Public Health has "another string to his bow," because many appointments are thus open to him which otherwise would not be available for him. Finally, there is no doubt that a medical officer of health who has tact, patience, and energy has the opportunity of following a career full of unceasing interest for himself and of rendering most valuable services to the community.

